# Pulmonary Function Tests as Predictors of Successful Tracheostomy Decannulation: A 60-Day Prospective Study

**DOI:** 10.7150/ijms.126157

**Published:** 2026-05-29

**Authors:** Chin-Chia Yang, Wen-Lin Yu, Meng-Heng Hsieh, Ching-Tzu Huang, Mei-Hsiu Chuang, Hsing-Yu Chen

**Affiliations:** 1 Department of Chinese Internal Medicine, Center for Traditional Chinese Medicine, Chang Gung Memorial Hospital, Taoyuan, Taiwan.; 2 Department of Thoracic Medicine, Chang Gung Memorial Hospital, Linkou, Taiwan.; 3 Department of Respiratory Therapist, Chang Gung Memorial Hospital, Linkou, Taiwan.; 4 Department of Respiratory Therapist, Chang Gung Memorial Hospital, Taoyuan, Taiwan.; 5 School of Traditional Chinese Medicine, College of Medicine, Chang Gung University, Taoyuan, Taiwan.

**Keywords:** tracheostomy, decannulation, pulmonary function test, forced vital capacity, peak expiratory flow rate

## Abstract

**Background:**

Early tracheostomy decannulation prevents complications, but premature removal poses risks. While various methods exist to assess readiness, the predictive role of pulmonary function tests remains unclear.

**Methods:**

This exploratory prospective observational study enrolled 46 patients at Chang Gung Memorial Hospital, Taoyuan, from March 2020 to December 2023. Participants underwent pulmonary function and coughing ability assessments—including forced vital capacity (FVC), peak expiratory flow rate (PEFR), maximum expiratory pressure (MEP), and oxygen saturation (SpO_2_)—prior to decannulation. Success was defined as no reinsertion within 60 days. Logistic regression analysis and receiver operating characteristic (ROC) curve analysis were performed.

**Results:**

Factors associated with a successful outcome were younger age, male sex, strong cough function, no nasogastric tube, and higher albumin levels. Key respiratory-function metrics (higher MEP, PEFR, FVC, and SpO_2_) were significantly linked to successful decannulation. FVC (≥ 1.35 L) and PEFR (≥ 95 L/min) strongly predicted success, with a combined metric (FVC + PEFR + MEP) achieving the highest accuracy.

**Conclusion:**

For patients with an average tracheostomy duration of > 300 days, FVC, PEFR and MEP are feasible predictors of successful decannulation. Further studies are needed to validate these results.

## Introduction

Tracheotomy is the surgical creation of an opening in the trachea, performed to assist patients with prolonged mechanical ventilation, relieve acute upper-airway blockages when intubation is not possible, manage chronic upper-airway obstructions, and prepare for significant head and neck surgeries^1^, ^2^. However, if the upper airways are bypassed by the tracheotomy for too long, the patient may be unable to speak, which may substantially diminish their quality of life, hamper their physical recovery, and even result in anxiety and depression^3^. Therefore, timely decannulation of a tracheostomy can improve a patient's quality of life, including their facial appearance, swallowing function, communication ability, and social reintegration. However, no consensus regarding the optimal timing for tracheostomy decannulation or the acceptable duration of tracheostomy placement has been reached^4^-^6^. In one study, the average number of days that patients with a tracheostomy were maintained on a ventilator was 70.6 days, and the average number of days that they were maintained on a respirator was 37.2 days^7^. In a study of 214 patients, the average time before decannulation ranged from 8 days to 46 months^8^. Delayed decannulation can cause tracheal stenosis, suprastomal granulation tissue, tracheomalacia, recurrent pneumonia, tracheoarterial fistula, tracheoesophageal fistula, and dysphagia^2^, ^8^, ^9^. Consequently, tracheostomy may complicate daily patient care and impair quality of life^1^, ^10^, ^11^. However, premature removal of a tracheostomy tube can also result in severe complications, such as a prolonged intensive care unit stay, readmissions, and higher mortality rates^4^, ^12^. Therefore, determining the optimal timing for tube removal has become a crucial issue for respiratory care. This issue is particularly important for respiratory therapists, who play a central role in airway management, ventilator weaning, and ensuring patient safety during decannulation.

Despite the fact that decannulation is a crucial aspect of clinical and home care for patients with tracheostomies, definite predictors of successful removal of the tracheostomy tube remains inconclusive^12^-^14^. Parameters commonly used to assess decannulation readiness include clinical condition, airway patency, swallowing ability, consciousness, and airway secretion clearance^13^. According to the consensus statement of the American Academy of Otolaryngology—Head and Neck Surgery, tracheostomy capping should be tolerated for 12, 24, or 48 hours with careful monitoring for possible respiratory distress before decannulation^15^. A study has also shown that the ability to tolerate a speaking valve for 4 hours can be an indicator of successful decannulation^7^, ^16^. Clinically, the patient's coughing ability can be evaluated by assessing the maximum expiratory pressure (MEP)^12^, ^17^, ^18^, peak cough flow^19^, ^20^, and peak expiratory flow rate (PEFR)^7^, ^12^. These indicators are typically quite complex and require a long time to comprehensively evaluate^7^. On the other hand, the overall pulmonary function is important for keeping airway patency^21^, and this function could be qualitatively determined during clinical examination via tracheostomy capping^16^, corking, spigotting, or downsizing to smaller tubes. However, no studies have focused on the importance of quantitative pulmonary function testing in patients planning to remove tracheostomy tube, despite the fact that lung capacity and expansion may be crucial in ventilator weaning^22^, ^23^.

The aim of this study was to evaluate the associations between coughing ability and/or pulmonary function and successful tracheostomy decannulation of using an exploratory prospective observational study design. We anticipated that the results would enhance our understanding of various methods of assessing decannulation readiness and demonstrate the importance of respiratory function in decision-making. Ultimately, this knowledge can strengthen evidence-based practice and support respiratory care professionals in making safer and more effective decannulation decisions.

## Methods

### Study population and protocol

We performed this exploratory prospective observational study to identify the best indicator for decannulation during and after the coronavirus pandemic era. We collected data from patients treated with a tracheostomy at the Taoyuan branch of Chang Gung Memorial Hospital from 1 March 2020 to 31 December 2023. In alignment with recent literature for multidisciplinary and protocolized care, we implemented a standardized decannulation framework designed to enhance patient safety and procedural effectiveness^12^, ^16^, ^24^. Patients were evaluated for decannulation readiness once the primary reason necessitating the tracheostomy had stabilized by doctors and nurses. Initial clearance was obtained from the Otorhinolaryngology (ENT) service, which confirmed upper airway patency via fiberoptic bronchoscopy and ruled out significant subglottic stenosis, edema or granulation tissue. Concurrently, chest X-rays were also reviewed to ensure no significant infiltrates or air trapping. Clinical stability was determined by pulmonologists (Dr. Hsieh) according to his clinical experience, such as SpO2 > 92% on minimal oxygen support, spontaneous cough ability, and manageable secretions. Eligible patients were then admitted to the ICU for a 24-hour tracheostomy capping trial. Prior to trial, the tracheostomy cuff was fully deflated to facilitate airflow through the upper airway. Patients were monitored closely for signs of respiratory distress, including tachypnea (respiratory rate > 25 breaths/min), use of accessory muscles, or subjective dyspnea. Patients who successfully tolerated the 24-hour capping period without physiological compromise were eligible for inclusion in the study. The exclusion criteria were as follows: (1) pregnant or currently breastfeeding; (2) inability to cooperate with testing and verbal command; (3) unstable vital signs; and (4) transferal to another hospital. After the pulmonologist or otolaryngologist confirmed that eligible patients were eligible for decannulation and the patients had given their informed consent, respiratory therapists were consulted and the patients' coughing ability and pulmonary function of eligible patients were tested. The tracheostomy tube was removed within 48-96 hours of the collection of respiratory parameters. The decision for decannulation was determined by pulmonologists, and the pulmonary function tests were performed only after the decannulation decision had been reached. Consequently, the test results remained independent of the clinical determination for tube removal. The protocol of this study was approved by the institutional review board of the Chang Gung Medical Foundation (IRB no.: 202000095B0C501). This study was also registered in the ISRCTN registry (reference number: 16452082).

### Outcome definition

The outcome of our study was the identification of factors predictive of successful decannulation within a 60-day follow-up period. Participants who were decannulated within three days of their initial assessment and had a stable respiratory condition and no recannulation during the follow-up period were defined as having been successfully decannulated. As in previous studies, a period of 72 hours was chosen as the initial time frame to assess whether recannulation was needed^25^. However, a short observation period may not be sufficient to reflect the long-term stability of decannulation, as some patients may experience delayed respiratory complications. Therefore, to strengthen the methodological rigor of our study and to more comprehensively evaluate patient prognosis, we extended the follow-up period to 60 days. For this reason, the need for recannulation, within either three or 60 days of removal, was defined as a decannulation failure.

### Covariates

We collected data on age, sex, smoking habits, cooperation (i.e., the ability to follow instructions), and underlying conditions (diabetes, hypertension, congestive heart failure, COVID-19, chronic obstructive lung disease, pneumonia, hepatitis, malignancies, cerebrovascular accident, trauma, and surgery). We tracked each patient's comorbidity history up to one year prior to admission. Additionally, we recorded their latest biochemical profile (albumin concentration, alanine transaminase concentration, white blood cell count, hemoglobin concentration, platelet count, blood urea nitrogen concentration, creatinine concentration, sodium concentration, and potassium concentration) and body mass index (BMI) before testing their coughing ability and pulmonary function as baseline biochemical data.

We also recorded the following covariates related to coughing ability and pulmonary function: interval between tracheostomy insertion and testing; presence of airway polyps; excess airway secretions (defined as suctioning more than twice within 8h); strong cough function (defined as being able to mobilize secretions to the oropharynx for expectoration^5^, ^26^); and use of a nasogastric tube. To objectively assess coughing ability, participants were positioned in a high Fowler's position (90 degrees elevation) assessed at the bedside by a respiratory therapist or a pulmonologist. A 'strong' cough was recorded if secretions were visibly or audibly moved from the lower airway to the oropharynx, allowing for spontaneous expectoration or easy clearance via oral suctioning. We also tested pulmonary function objectively using the following parameters: rapid shallow breathing index (RSBI), minute ventilation (MV), tidal volume (V_T_), respiratory rate (RR), heart rate (HR), oxygen saturation (SpO_2_), and forced vital capacity (FVC). The PEFR was measured using the peak flow meter (TruZone; Monaghan Medical Corp; Plattsburgh, NY.), whereas the V_T_, and FVC were measured with the wright respirometer (Halo/scale Wright respirometer standard type; nSpire Health; Hertford, UK). MIP and MEP were measured using a gas pressure gauge (MTC Gas Pressure Gauge; Meditech Trading Co., Ltd.; New Taipei City, Taiwan). The RSBI and MV were calculated by dividing and multiplying the RR by the V_T_, respectively. During the measurements, all patients were positioned in a high Fowler's position with the tracheostomy tube capped to optimize diaphragmatic excursion. The respiratory therapists provided standardized verbal coaching, instructing patients to 'take the deepest breath possible, hold for 1 second, and then blast the air out as hard and fast as possible'. To ensure maximal effort, a minimum of three acceptable trials^27^ were performed for each parameter, with at least one minute of rest between trials to prevent respiratory muscle fatigue. The maximum value among the three reproducible maneuvers was recorded for analysis. FVC, PEFR, MIP, and MEP were chosen as our primary predictors because they collectively assess different facets of respiratory mechanics essential for successful decannulation. FVC reflects overall lung volume and the patient's ventilatory reserve^28^, ^29^; PEFR serves as a dynamic measure of expiratory flow and cough intensity^30^; MIP was a sensitive measure of respiratory muscle strength in neuromuscular disease^31^; and MEP provides not only a static measure of expiratory muscle strength but also a common predictor in previous studies^12^, ^17^, ^18^, ^32^. Besides, we utilized the Wright respirometer, portable peak flow meter, and gas pressure gauge due to their high reliability and availability in our hospital.

### Statistical analysis

To compare patients' baseline characteristics, coughing ability, and pulmonary function, we used the chi-square test or Fisher's exact test for categorical variables, as appropriate, and an independent Student *t*-test for continuous variables. We then conducted logistic regression analysis to calculate the odds ratio (OR) for tracheostomy removal based on different variables. Successful removal was set as the dependent variable, and all coughing ability (MIP and MEP) and respiratory function (RSBI, MV, V_T_, RR, HR, SpO_2_, PEFR, and FVC) values were used as independent variables in the logistic regression model. We calculated the area under the receiver operating characteristic (ROC) curve (AUC) to determine the predictive value of single parameters for 60-day successful tracheostomy removal, and we used Youden's *J* statistic to calculate potential optimal cut-off values for single candidate parameters. Subgroup analyses will be executed to examine the predictive performance (AUC) across different lengths of stay. The cohort will be stratified by the median tracheostomy duration, allowing for a comparative assessment of the model's discriminative powers between patients with relatively shorter versus longer tracheostomy durations. Besides, we used Pearson's correlation and a correlation matrix to calculate the linear relationship between all pairs of covariates and thus determine whether different parameters could feasibly be combined. Finally, we evaluated single and combined candidate parameters for prediction of successful removal of tracheostomy in 60 days via C-statistics with 95% confidence intervals (CIs). All statistical analyses were performed using the commercial statistical software program, STATA (release 17; StataCorp, College Station, TX). A p-value less than 0.05 was considered statistically significant.

## Results

### Demographic and clinical features

**Figure 1:**
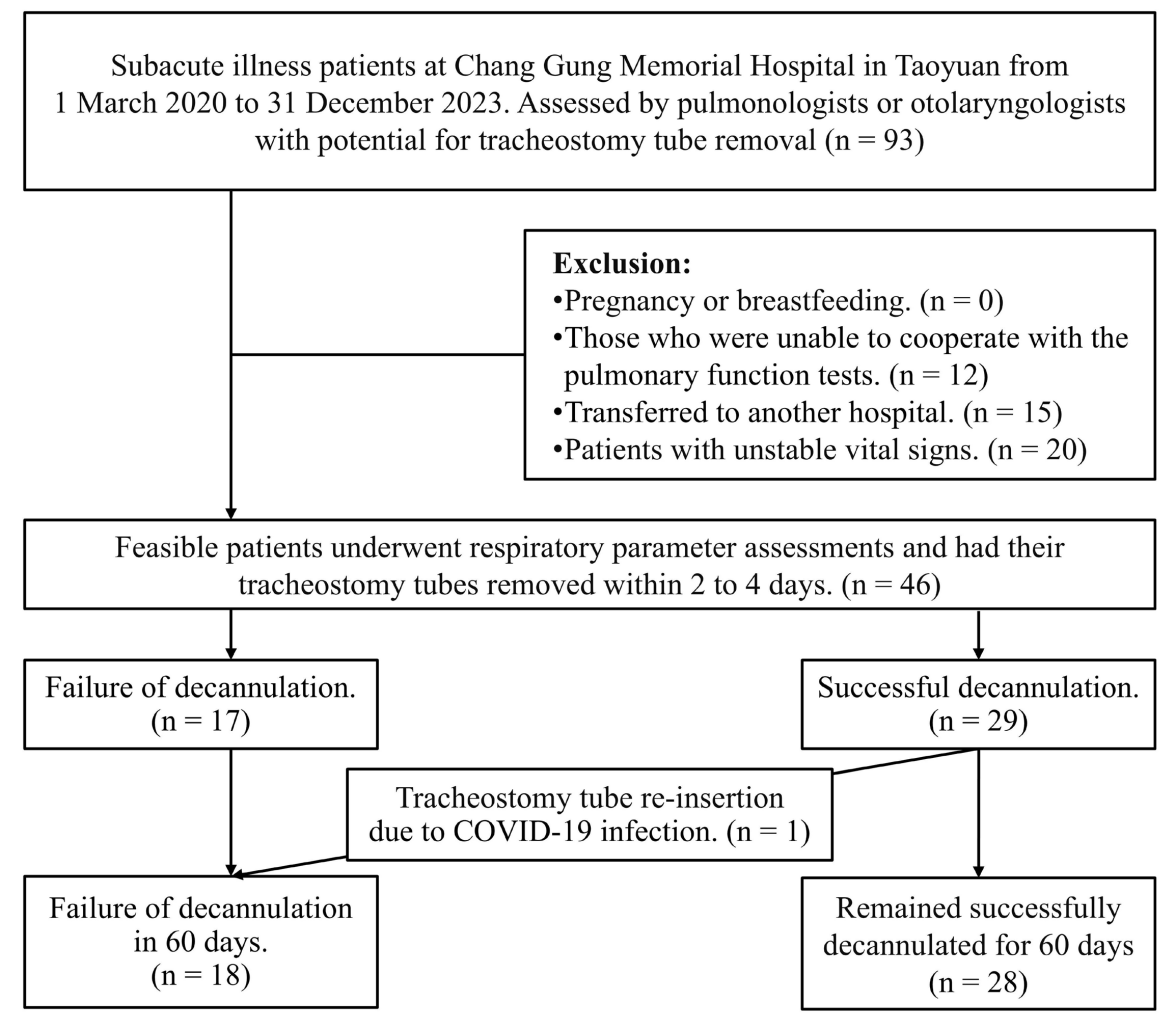
Flow diagram of this study.

During the study period, 46 participants were enrolled, with 28 individuals in the successful decannulation group and 18 in the failed decannulation group as shown in Figure 1. The demographic and clinical features of both groups are listed in Table 1. Significant differences were observed between the two groups in terms of sex and age. The mean age was younger in the successful decannulation group (53.68 years vs. 62.94 years, p = 0.044) and the proportion of males was higher in the successful decannulation group than in the failed decannulation group (92.9% vs. 66.7%, p = 0.022). However, no significant differences between the two groups were observed in terms of smoking history or BMI. Regarding underlying conditions, no differences in the presence of heart failure, chronic obstructive pulmonary disease, COVID-19, or cerebrovascular accidents were observed between the two groups. Conversely, the prevalence of malignancy was higher in the failed decannulation group than in the successful decannulation group (22.2% vs. 3.6%, p = 0.047). In terms of biochemical profiles, only two significant differences were observed between the two groups. The successful decannulation group had a higher albumin level than the failed decannulation group (3.79 ± 0.54 g/dL vs. 3.09 ± 0.97 g/dL, p = 0.018). The successful decannulation group also had a higher platelet count (286,740 ± 88,060/μL vs. 230,060 ± 73,920/μL, p = 0.037).

### Association between respiratory function and tracheostomy removal

As shown in Table 2, the time interval from tracheostomy insertion to testing did not differ significantly between the successful and failed decannulation groups (242.71 ± 312.33 days vs. 395.33 ± 380.10 days, p = 0.14). The successful decannulation group had a higher proportion of individuals with strong cough function (85.7% vs. 50.0%, p = 0.009) and a lower proportion who needed a nasogastric tube (35.7% vs. 72.2%, p = 0.016). Regarding the coughing ability and respiratory function tests, the successful decannulation group had a higher MEP (68.64 cmH_2_O vs. 49.44 cmH_2_O, p = 0.017), SpO_2_ (97.68% vs. 96.89%, p = 0.038), PEFR (161.43 L/min vs. 79.44 L/min, p < 0.001), and FVC (2.09 L vs. 0.92 L, p < 0.001) than the failed decannulation group.

The results of the logistic regression analysis are presented in Table 3. Of the demographic and clinical features analyzed, male sex, strong cough function, and absence of a nasogastric tube remained associated with successful tracheostomy removal. In terms of the objective respiratory function measures, a 1 cmH_2_O elevation in MEP was associated with 3% higher odds of successful removal (OR: 1.03; 95% CI: 1.00-1.06; p = 0.028). In terms of pulmonary function, a higher FVC (OR: 9.27; 95% CI: 2.55-33.77; p = 0.001) and PEFR (OR: 1.03; 95% CI: 1.01-1.04; p = 0.001) were significantly associated with a higher probability of decannulation without complications within 60 days. That is, the rate of successful removal may increase approximately ninefold with every 1 L increase in FVC and by 3% with every 1 L/min increase in PEFR.

**Figure 2:**
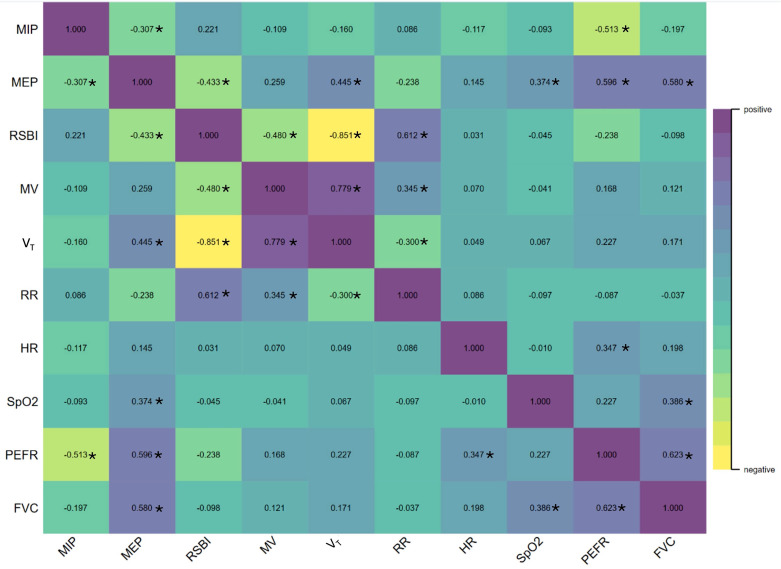
** Correlations between potential indicators for successful tracheostomy removal in 60 days.** The numbers in the figure represent correlation coefficients between the two corresponding indicators, and * indicates p < 0.05. The FVC was moderately correlated with the MEP (r = 0.580, p < 0.001) and PEFR (r = 0.623, p < 0.001). A strong positive correlation was noted between the MV and V_T_ (r = 0.779). Abbreviations: FVC, forced vital capacity; HR, heart rate; MIP, maximum inspiratory pressure; MEP, maximum expiratory pressure; MV, minute ventilation; PEFR, peak expiratory flow rate; RR, respiratory rate; RSBI, rapid shallow breathing index; SpO_2_, oxygen saturation; V_T_, tidal volume.

The relationships between the potential predictors in our study are summarized in the correlation matrix map shown in Figure 2. The FVC was weakly correlated with the RR, HR, SpO_2_, V_T_, MV, RSBI, and MIP, and moderately correlated with the MEP (*r* = 0.580, p < 0.001) and PEFR (*r* = 0.623, p < 0.001). A strong negative correlation was observed between the RSBI and V_T_ (*r* = -0.851), and a strong positive correlation between the MV and V_T_ (*r* = 0.779).

### ROC curves and cut-off values of respiratory parameters

**Figure 3:**
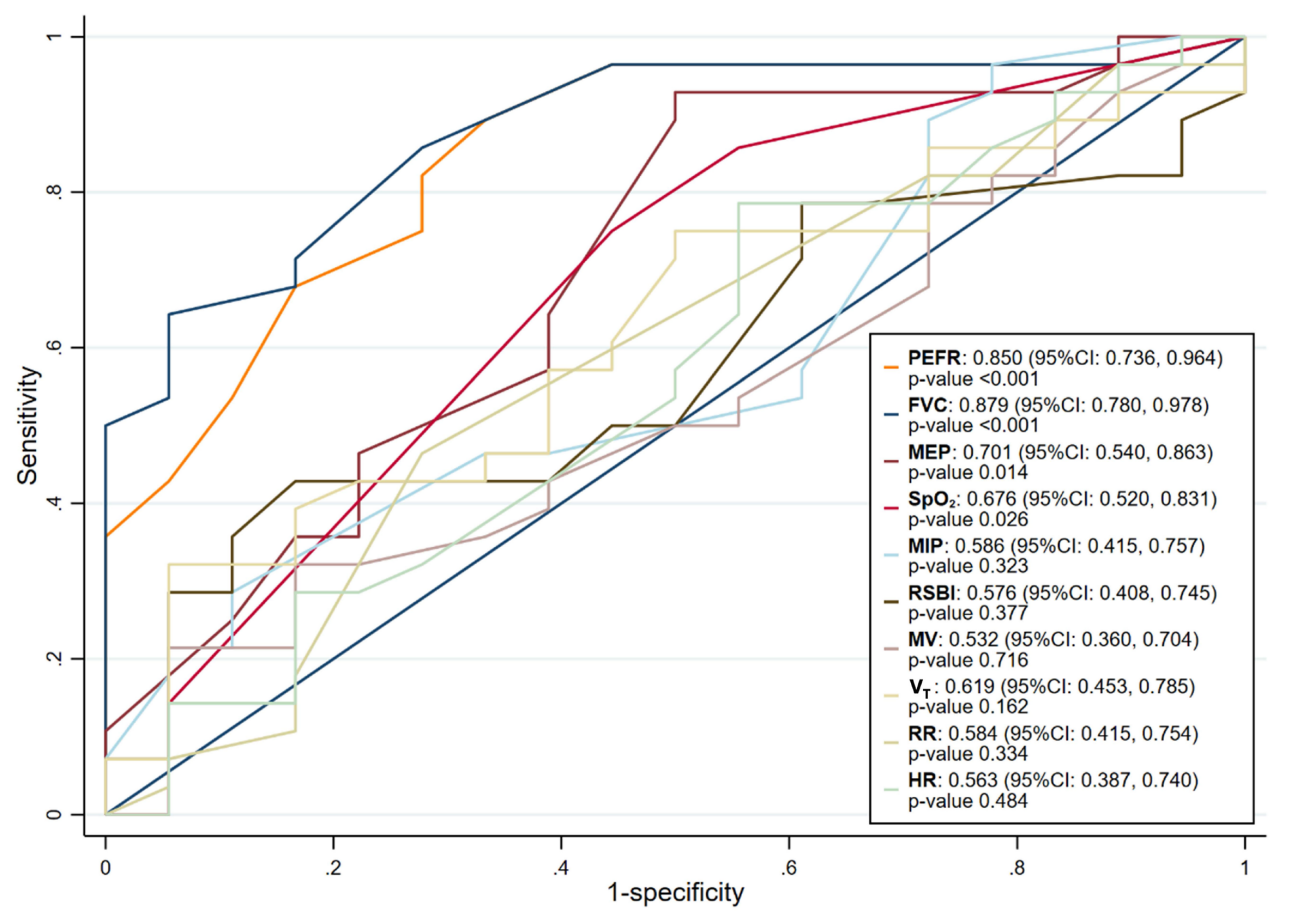
** ROC curves of single parameters for successful tracheostomy removal in 60 days.** PEFR and FVC had the highest AUCs for single parameters. The AUC of the PEFR for predicting successful decannulation was 0.850 (95% CI: 0.736-0.964; p < 0.001) and that of the FVC was 0.879 (95% CI: 0.780-0.978; p < 0.001). Abbreviations: AUC, area under the curve; FVC, forced vital capacity; HR, heart rate; MIP, maximum inspiratory pressure; MEP, maximum expiratory pressure; MV, minute ventilation; PEFR, peak expiratory flow rate; RSBI, rapid shallow breathing index; RR, respiratory rate; SpO_2_, oxygen saturation; V_T_, tidal volume.

As shown in Figure 3, PEFR and FVC had the highest AUCs for single parameters. The AUC of the PEFR for predicting successful decannulation was 0.850 (95% CI: 0.736-0.964; p < 0.001) and that of the FVC was 0.879 (95% CI: 0.780-0.978; p < 0.001). The AUCs for MEP (0.701; 95% CI: 0.540-0.863; p = 0.014) and SpO_2_ (0.676; 95% CI: 0.520-0.831; p = 0.026) were lower than those for PEFR and FVC. Upon subgroup analysis by median tracheostomy duration (202 days), the values of AUC remained within acceptable ranges, which indicated the usefulness of these two factors in population with shorter tracheostomy duration (Table 4). The optimal threshold for PEFR was 95 L/min (sensitivity 89%, specificity 67%), whereas that for FVC was 1.35 L (sensitivity 64%, specificity 94%). Based on these cut-off values, PEFR had a positive predictive value (PPV) of 0.806 and a negative predictive value (NPV) of 0.797, and its accuracy was 0.804. The PPV, NPV, and accuracy of FVC were 0.943, 0.627, and 0.757, respectively. Our findings suggest that a PEFR ≥ 95 L/min and FVC ≥ 1.35 L increased the odds of successful decannulation approximately 16.67 times (OR: 16.67; 95% CI: 3.55-78.32) and 15.6 times (OR: 15.60; 95% CI: 3.56-68.39), respectively (Table 5).

### Combined coughing ability and pulmonary function tests yielded the best results

**Figure 4:**
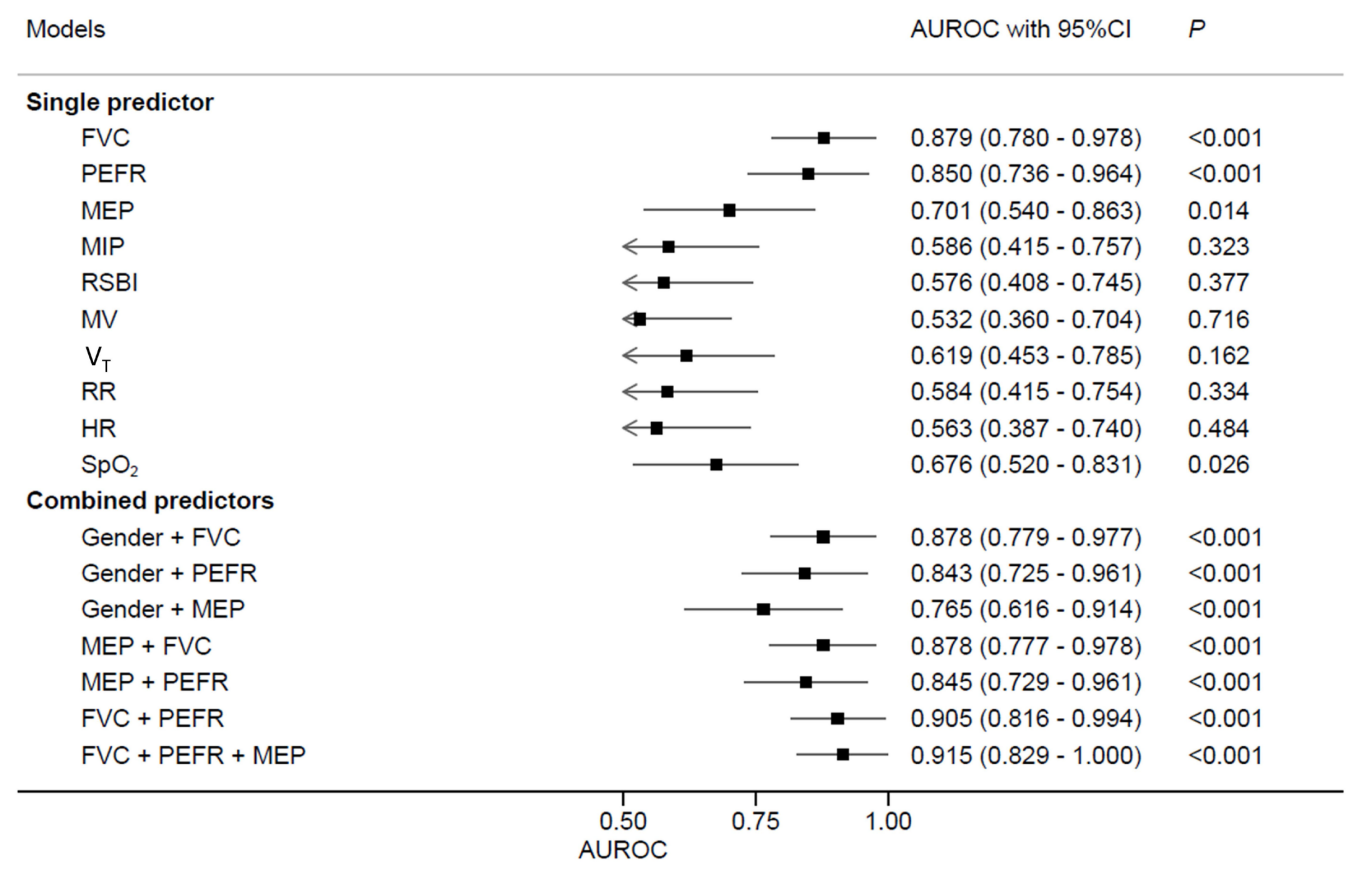
** Area under the curve for various models involving single and combined parameters.** Regarding single predictors, FVC and PEFR exhibited higher AUC values (0.879 and 0.850, respectively). However, among combined candidate predictors, the models FVC + PEFR + MEP and FVC + PEFR showed highest and second highest AUC values of 0.915 (95% CI: 0.829-1.000, p-value < 0.001) and 0.905 (95% CI: 0.816-0.994, p-value < 0.001), respectively. Abbreviations: AUC, area under the curve; FVC, forced vital capacity; HR, heart rate; MIP, maximum inspiratory pressure; MEP, maximum expiratory pressure; MV, minute ventilation; PEFR, peak expiratory flow rate; RSBI, rapid shallow breathing index; RR, respiratory rate; SpO_2_, oxygen saturation; V_T_, tidal volume.

In terms of single predictors, FVC and PEFR had the highest AUCs (0.879 and 0.850, respectively). However, in terms of combined candidate predictors, the FVC + PEFR + MEP and FVC + PEFR models exhibited the highest and second highest AUCs: 0.915 (95% CI: 0.829-1.000; p < 0.001) and 0.905 (95% CI: 0.816-0.994; p < 0.001), respectively (Figure 4).

## Discussion

This was the first study on the feasibility of using pulmonary function tests to predict successful decannulation, and it yielded positive results. Specifically, a FVC ≥ 1.35 L and PEFR ≥ 95 L/min may serve as reliable criteria for the successful decannulation of patients who have had a tracheostomy tube in place for an extended period (average: 302.4 days). Further, the use of pulmonary function tests and coughing ability tests combined may provide the highest predictive value for successful decannulation, particularly when MEP is combined with PEFR and FVC. Notably, only one patient from the successful decannulation group required reintubation 60 days after decannulation, owing to a COVID-19 infection. As a result, this patient was ultimately reclassified into the failure group.

Recent multicenter analyses from international collaborative databases, including the Global Tracheostomy Collaborative (GTC), have established that decannulation outcomes are governed by multidimensional determinants such as neurologic status, secretion burden, swallowing competence, ventilatory dependence, and institutional practices^33^. A fundamental distinction between the present study and most studies lies in patient chronicity, in which most studies predominantly reflected acute or subacute trajectories (median tracheostomy durations typically ranging from 13 to 28 days)^11^, ^33^. In contrast, our cohort represents a chronic illness phenotype, characterized by a mean cannulation interval of 302.4 days. In this chronic phase, predictors primarily linked to the resolution of the precipitating critical illness may be insufficient for precise bedside decision-making, as barriers shift toward limitations in physiological reserve, respiratory muscle deconditioning, and secretion clearance capacity. Our findings offer a complementary physiological perspective to existing epidemiological risk-stratification frameworks.

On the other hand, MEP serves a commonly used indicator of coughing ability and has been identified in multiple studies as a predictor of successful decannulation^12^, ^17^, ^18^, ^34^. A systematic review showed that an MEP > 40 cmH_2_O is a predictor of successful decannulation^12^. However, in our study, MEP was not the best single predictor. Although MEP serves as a valuable measure of cough strength, a comprehensive assessment of respiratory function must consider the performance of the entire respiratory system, which can be achieved through detailed pulmonary function testing.

In our study, the pulmonary function tests, especially PEFR and FVC, were more strongly associated with successful decannulation than MEP, which indicates coughing ability. An effective cough requires inspiration to above 80% of total lung capacity, glottic closure, and active exhalation with maximal expiratory flow coordinated with expulsive opening of the glottis. Consequently, cough efficacy depends on both inspiratory and expiratory capacities, as well as the strength of the inspiratory and expiratory muscles^35^, ^36^. PEFR is a straightforward and noninvasive measurement that depends on voluntary effort, expiratory muscle strength, airway resistance, and the elastic recoil of the lungs^32^. To date, few studies on the relationship between PEFR and decannulation have been conducted^7^, ^12^, ^37^. In one prospective data from East Asian cohorts, PEFR was used as a measure of cough intensity, and the findings suggested that a PEFR of > 1.67 L/s (about 100 L/min) indicates sufficient cough ability for decannulation^7^. This proposed cutoff appears lower than thresholds reported in Western populations, where higher PEFR have often been associated with successful extubation outcomes^38^. Such differences may partially reflect anthropometric variations between Asian and Western cohorts, including generally smaller body size, shorter average stature, and lower absolute lung volumes in East Asian populations^39^. Furthermore, the resistance imposed by the tracheostomy tube itself during measurement may underestimate the patient's true capacity, as values often increase by approximately 15-20% post-decannulation^30^, ^40^. This phenomenon is physiologically plausible because the artificial airway reduces effective airway diameter, increases turbulent flow, and bypasses normal glottic modulation, all of which attenuate measured expiratory flows. While other studies have proposed a higher threshold of > 160 L/min to ensure airway patency^12^, ^37^, ^41^, these values were largely derived from measurements obtained via the natural airway^42^. Many studies supporting a > 160 L/min criterion measure cough peak flow at the mouth after tracheostomy tube removal or under relatively unobstructed upper-airway conditions, whereas PEFR measured through a tracheostomy tube (even with a speaking valve) is systematically lower due to additional airflow resistance and the altered upper-airway mechanics. Given that cough effectiveness depends on adequate inspiratory volume, glottic competence, and coordinated expiratory muscle activation^43^, absolute PEFR or CPF cut-offs are not directly interchangeable across studies with differing airway configurations and measurement techniques. Recent work in prolonged tracheostomy patients has also supported a lower cough-flow threshold (around 100 L/min) when measured with the tracheostomy tube and speaking valve in place, reinforcing the concept that technique-specific cut-offs are necessary^7^, ^19^, ^44^. A fundamental distinction of our study is the chronicity of the cohort, with a mean cannulation duration of 302.43 days, representing a chronic critical illness phenotype. In such patients, prolonged tracheostomy and critical illness contribute to respiratory muscle atrophy and deconditioning, impairing both inspiratory capacity and expiratory flow generation—key components of effective cough^45^, ^46^. Therefore, higher thresholds such as >160 L/min^12^, ^37^, ^41^, largely derived from post-decannulation or oral measurements, may not be directly applicable to chronically cannulated patients. Our findings demonstrate that PEFR values as low as 95 L/min were still compatible with successful decannulation (except for one COVID-19-related case), suggesting that in patients with long-term tracheostomy and muscle atrophy, a lower but functionally sufficient PEFR should not, in isolation, justify delaying decannulation.

To the best of our knowledge, no studies on the use of FVC as a predictive parameter for decannulation have been published. FVC is an indicator used in pulmonary function testing to evaluate whether the lungs possess elastic recoil, and it can reflect breathing ability and ventilation capacity^47^-^49^. In patients with cervical spinal cord injuries, FVC has been used as a marker of swallowing function in those who undergo tracheostomy^50^. FVC is associated with the strength of the inspiratory muscles^28^, particularly the diaphragm, which is innervated by the phrenic nerve, which originates from the C3-C5 spinal nerve roots. The interplay between swallowing and breathing may have affected our results, since precise coordination between the two is crucial to prevent pulmonary aspiration^29^. Swallowing function is thus also an important factor in evaluating the success of decannulation^51^.

After investigating the use of single parameters, we found that combining multiple parameters provided the highest predictive values. Considering parameters such as PEFR and FVC along with MEP may provide a more comprehensive but accessible approach to evaluating decannulation readiness. We found that the combinations FVC + PEFR and FVC + PEFR + MEP had the second highest and highest AUC values, respectively (0.905 and 0.915). Since FVC is an indicator of the maximal strength of the inspiratory and expiratory muscles, PEFR reflects dynamic respiratory muscle function, and MEP indicates static respiratory muscle function^52^, the combination of the three can be used to assess a patient's recovery in terms of respiratory function, coughing ability, and swallowing function. This combined model may serve as the most effective predictor of successful decannulation.

Still, this study has several limitations. First, the modest sample size increases the risk of statistical overfitting and also limits the factors included in the regression model considering model stability. To address the importance of pulmonary function test parameters, the inclusion of patients with varied etiologies, including trauma, CVA, and malignancy, introduces potential confounding that was not fully incorporated into the multivariable regression model, which may cause potential bias on statistical estimation. Second, to facilitate data collection in a clinical setting, nasogastric (NG) tube placement was utilized as a surrogate marker for dysphagia, and the ability to follow verbal commands was used as a pragmatic inclusion criterion rather than comprehensive neurological scales (e.g., NIHSS or GCS). We acknowledge that these proxy measures may lack the sensitivity of standardized assessments and could potentially introduce residual confounding, thereby influencing the estimated effect sizes in the regression analysis. However, these indicators reflect real-world clinical documentation during the study period, and the influence would be minimal. Third, the mean duration of tracheostomy in this study was approximately 300 days, reflecting a chronic weaning population. Although subgroup analysis suggested that PEFR and FVC remained feasible predictors for patients with a duration of <200 days, their discriminative power may vary in acute or subacute settings where durations are substantially shorter. This discrepancy highlights the potential influence of chronicity on respiratory muscle performance metrics. Fourth, the small cohort may limit the generalizability of the results to broader populations. Therefore, further validation in larger, multicenter cohorts is essential to confirm these thresholds and ensure their clinical applicability with adequate power of multivariable adjustment. Additionally, pulmonary function testing requires patient cooperation and the ability to follow instructions given by respiratory therapists. These tests cannot be used on patients who are unconscious, and that is precisely the group that most urgently requires objective assessments to determine the feasibility of decannulation. Other assessment methods are therefore still needed. Finally, Asians generally have a lower BMI than Europeans and Americans, which may affect optimal pulmonary function values. Therefore, more research is needed to standardize these values for Asians. Similarly, the generalizability of our findings regarding the use of PEFR and FVC for non-Asian patients may be questionable. While the proposed cut-off values for FVC and PEFR are statistically derived and internally consistent, their clinical interpretation requires caution. These measures should serve as adjuncts to multidisciplinary decision-making rather than stand-alone criteria to avoid the risk of over-interpreting these values as definitive decision rules.

## Conclusions

For patients with an average duration of tracheostomy of more than 300 days, our study demonstrated the feasibility of using FVC and PEFR to predict successful decannulation. We also identified optimal cut-off values for FVC and PEFR. Further studies are needed to validate these results.

## Figures and Tables

**Table 1:** Demographic and clinical features of enrolled participants.

		Total (n = 46)	Failed decannulation (n = 18)	Successful decannulation (n = 28)	p
Age, years		57.30 (15.34)	62.94 (12.89)	53.68 (15.90)	**0.044**
Male sex, n		38 (82.6%)	12 (66.7%)	26 (92.9%)	**0.022**
BMI, kg/m^2^		22.33 (4.34)	22.79 (5.34)	22.03 (3.63)	0.57
	< 18.5 (underweight), n	9 (19.6%)	2 (11.1%)	7 (25.0%)	0.27
	18.5-24.9 (healthy weight), n	24 (52.2%)	12 (66.7%)	12 (42.9%)	
	≥ 25 (overweight), n	13 (28.3%)	4 (22.2%)	9 (32.1%)	
Smoking, n		13 (28.3%)	6 (33.3%)	7 (25.0%)	0.54
Cooperation, n		40 (87.0%)	16 (88.9%)	24 (85.7%)	0.76
Comorbidities					
	DM, n	15 (32.6%)	5 (27.8%)	10 (35.7%)	0.58
	Hypertension, n	21 (45.7%)	8 (44.4%)	13 (46.4%)	0.90
	CHF, n	2 (4.3%)	1 (5.6%)	1 (3.6%)	0.75
	COVID-19, n	9 (19.6%)	5 (27.8%)	4 (14.3%)	0.26
	COPD, n	3 (6.5%)	1 (5.6%)	2 (7.1%)	0.83
	Pneumonia, n	20 (43.5%)	6 (33.3%)	14 (50.0%)	0.27
	Hemodialysis, n	4 (8.7%)	2 (11.1%)	2 (7.1%)	0.64
	Hepatitis, n	1 (2.2%)	1 (5.6%)	0 (0.0%)	0.21
	Malignancies, n	5 (10.9%)	4 (22.2%)	1 (3.6%)	**0.047**
	CVA, n	13 (28.3%)	4 (22.2%)	9 (32.1%)	0.47
	Trauma, n	14 (30.4%)	3 (16.7%)	11 (39.3%)	0.10
	Surgery, n	29 (63.0%)	10 (55.6%)	19 (67.9%)	0.40
Biochemical profiles					
	Albumin, g/dL	3.49 (0.82)	3.09 (0.97)	3.79 (0.54)	**0.018**
	ALT, U/L	35.68 (23.44)	40.31 (25.15)	32.72 (22.30)	0.32
	WBC, /µL	7.91 (2.59)	8.44 (2.98)	7.57 (2.32)	0.29
	Neutrophils, /µL	5.30 (2.28)	6.10 (2.57)	4.79 (1.97)	0.064
	Lymphocytes, /µL	1.79 (0.73)	1.55 (0.61)	1.94 (0.76)	0.087
	Hgb, g/dL	11.63 (2.33)	11.22 (2.45)	11.87 (2.27)	0.38
	Platelets, 1000/µL	265.65 (86.72)	230.06 (73.92)	286.74 (88.06)	**0.037**
	BUN, mg/dL	20.63 (19.09)	23.68 (25.69)	18.81 (14.09)	0.43
	Creatinine, mg/dL	1.21 (1.50)	1.41 (1.84)	1.08 (1.26)	0.49
	Na, mEq/L	138.05 (3.95)	136.59 (4.09)	138.96 (3.64)	0.051
	K, mEq/L	3.91 (0.58)	4.06 (0.81)	3.81 (0.36)	0.18

Abbreviations: ALT, alanine transaminase; BMI, body mass index; BUN, blood urea nitrogen; CHF, congestive heart failure; CVA, cerebrovascular accident; DM, diabetes mellitus; Hgb, hemoglobin; K, potassium; Na, sodium; WBC, white blood cells. Numbers presented are mean (standard deviation) and n (%). Values in bold are statistically significant (p < 0.05)

**Table 2:** Respiratory function test results.

	Total (n = 46)	Failed decannulation (n = 18)	Successful decannulation (n = 28)	p
Interval between tracheostomy insertion and test, days	302.43 (344.65)	395.33 (380.10)	242.71 (312.33)	0.14
Airway polyps, n	8 (17.4%)	4 (22.2%)	4 (14.3%)	0.49
Excessive airway secretion, n	18 (39.1%)	9 (50.0%)	9 (32.1%)	0.23
Strong cough function, n	33 (71.7%)	9 (50.0%)	24 (85.7%)	**0.009**
Use of NG tube, n	23 (50.0%)	13 (72.2%)	10 (35.7%)	**0.016**
MIP, cmH_2_O	-61.15 (21.90)	-56.28 (19.02)	-64.29 (23.35)	0.23
MEP, cmH_2_O	61.13 (27.03)	49.44 (26.37)	68.64 (25.10)	**0.017**
RSBI	42.15 (11.51)	44.00 (10.08)	40.96 (12.37)	0.39
MV, L/min	7.60 (1.71)	7.39 (1.66)	7.74 (1.76)	0.51
V_T_, mL	437.85 (114.21)	417.44 (97.13)	450.96 (123.87)	0.34
RR, times/min	17.52 (2.51)	17.78 (2.58)	17.36 (2.50)	0.58
HR, times/min	83.85 (9.35)	82.39 (9.97)	84.79 (8.99)	0.40
SpO_2_, %	97.37 (1.27)	96.89 (1.45)	97.68 (1.06)	**0.038**
PEFR, L/min	129.35 (71.81)	79.44 (53.96)	161.43 (63.52)	**< 0.001**
FVC, L	1.63 (0.94)	0.92 (0.58)	2.09 (0.83)	**< 0.001**

Abbreviations: FVC, forced vital capacity; HR, heart rate; MIP, maximum inspiratory pressure; MEP, maximum expiratory pressure; MV, minute ventilation; NG, nasogastric; PEFR, peak expiratory flow rate; RSBI, rapid shallow breathing index; RR, respiratory rate; V_T_, tidal volume. Numbers presented are mean (standard deviation) and n (%). Values in bold are statistically significant (p < 0.05).

**Table 3:** Logistic regression analysis of feasibility of tracheostomy removal in 60 days.

Parameter	β	SE	OR (95% CI)	p
Age, years				
≤ 40	reference			
41-60	-1.28	1.20	0.28 (0.03-2.90)	0.285
≥ 60	-1.70	1.16	0.18 (0.02-1.76)	0.141
Sex				
Female	reference			
Male	1.87	0.89	6.50 (1.14-37.05)	**0.035**
Respiratory function test results				
Strong cough function	1.79	0.72	6.00 (1.47-24.45)	**0.012**
Use of NG tube	-1.54	0.66	0.21 (0.06-0.78)	**0.019**
MIP	-0.02	0.02	0.98 (0.95-1.01)	0.229
MEP	0.03	0.01	1.03 (1.00-1.06)	**0.028**
RSBI	-0.02	0.03	0.98 (0.93-1.03)	0.381
MV	0.12	0.18	1.13 (0.79-1.61)	0.502
V_T_	0.00	0.00	1.00 (1.00-1.01)	0.337
RR	-0.07	0.12	0.93 (0.73-1.19)	0.577
HR	0.03	0.03	1.03 (0.96-1.10)	0.394
SpO_2_	0.52	0.26	1.68 (1.01-2.80)	0.046
PEFR	0.03	0.01	1.03 (1.01-1.04)	**0.001**
FVC	2.23	0.66	9.27 (2.55-33.77)	**0.001**

Abbreviations: CI, confidence interval; FVC, forced vital capacity; HR, heart rate; MIP, maximum inspiratory pressure; MEP, maximum expiratory pressure; MV, minute ventilation; NG, nasogastric; OR, odds ratio; PEFR, peak expiratory flow rate; RSBI, rapid shallow breathing index; RR, respiratory rate; V_T_, tidal volume. Values in bold are statistically significant (p < 0.05).

**Table 4:** Subgroup analysis on the AUC values of using FVC and PEFR to predict successful decannulation stratified by median interval between tracheostomy insertion and test (202 days).

	AUC	(95% CI)	p
PEFR (L/min)			
< 202 days	0.961	(0.892-1.000)	**<0.001**
≥ 202 days	0.848	(0.670-1.000)	**<0.001**
FVC (L)			
< 202 days	0.922	(0.773-1.000)	**<0.001**
≥ 202 days	0.731	(0.512-0.950)	**0.039**

Abbreviations: AUC, area under curve; CI, confidence interval; FVC, forced vital capacity; PEFR, peak expiratory flow rate. Values in bold are statistically significant (p < 0.05).

**Table 5:** Identification of PEFR and FVC cut-off values for successful tracheostomy removal with 60-day follow-up.

Parameter	β	SE	OR (95% CI)	p
PEFR (L/min)				
< 95	reference			
≥ 95	2.81	0.79	16.67 (3.55-78.32)	**< 0.001**
FVC (L)				
< 1.35	reference			
≥ 1.35	2.75	0.75	15.60 (3.56-68.39)	**< 0.001**

Abbreviations: CI, confidence interval; FVC, forced vital capacity; OR, odds ratio; PEFR, peak expiratory flow rate; SE, standard error. Values in bold are statistically significant (p < 0.05).

## Data Availability

Analyzed data are presented in the results of this manuscript, and raw data are not provided publicly.

## Abbreviations

ALT: alanine transaminase
AUC: area under the curve
BMI: body mass index
BUN: blood urea nitrogen
CHF: congestive heart failure
CI: confidence interval
COPD: chronic obstructive pulmonary disease
COVID-19: coronavirus disease of 2019
CVA: cerebrovascular accident
DM: diabetes mellitus
FVC: forced vital capacity
Hgb: hemoglobin
HR: heart rate
K: potassium
MEP: maximum expiratory pressure
MIP: maximum inspiratory pressure
MV: minute ventilation
Na: sodium
NG: nasogastric
OR: odds ratio
PEFR: peak expiratory flow rate
RR: respiratory rate
RSBI: rapid shallow breathing index
SE: standard error
SpO_2_: oxygen saturation
V_T_: tidal volume
WBC: white blood cells
